# Review: Anatomy, Descriptive and Surgical

**Published:** 1859-12

**Authors:** 


ART. VI. *Anatomy, Descriptive and Surgical.* By HENRY GRAY, F. R. S., Lecturer on Anatomy at St. George's Hospital. The Drawings by H. T. Carter, M. D., late Demonstrator of Anatomy at St. George's Hospital. The Dissections jointly by the Author and Dr. Carter. With three hundred and sixty-three engravings on wood. Philadelphia: Blanchard & Lea. 1859.

THERE is, at the present day, no lack of text-books upon human anatomy. Amongst these, there are two which, although differing considerably in design and scope, have generally been regarded as unexceptionable; we allude to the manual of Wilson, and the elaborate treatise of Sharpey & Quain. The first of these is the one most valued by the medical student, and perhaps, best adapted to his wants. It presents, within a narrow compass, a clear and concise exposition of anatomical science; and its faults are chiefly those of omission. The work of Sharpey & Quain, on the contrary, is distinguished for its completeness and accuracy of detail; and, in our opinion, is still the best and most comprehensive in the English language.

Notwithstanding, however, that our supply of elementary text-books appears ample, the work before us is one entitled to the highest praise, and we accordingly welcome it as a valuable addition to medical literature. Intermediate in fullness of detail between the treatises of Sharpey and of Wilson, its characteristic merit lies in the number and excellence of the engravings it contains. Most of these are original, of much larger than ordinary size, and admirably executed. The various parts are also lettered after the plan adopted in Holden's Osteology. It would be difficult to over-estimate the advantages offered by this mode of pictorial illustration. Bones, ligaments, muscles, bloodvessels, and nerves, are each in turn figured, and marked with their appropriate names; thus enabling the student to comprehend, at a glance, what would otherwise often be ignored, or at any rate, acquired only by prolonged and irksome application. As examples of the superiority of this manner of instruction, we may refer the reader to fig. 140, where he will find a faithful delineation of the muscles of the soft palate; or to figs. 58, 59, 60, and 61, illustrating the development of the sternum.

Cordially, however, as we approve of this, or of any other method that tends to facilitate the labors of the student, or to refresh the memory of the practitioner, we wish to be distinctly understood, that we do not think it calculated to supersede, in any degree, the necessity of thorough and diligent dissection. By this means alone can the student acquire that kind of knowledge which is indispensable to the practice of medicine or surgery; and it is a circumstance derogatory to the character of American Medical Schools, that dissection should be left at the option of the student, and a diploma granted to the man who has, perhaps, never in his life made the structure of the human body the subject of direct, personal examination. It is in view of this defect in our college requirements, and of the temptations which a book like Gray's Anatomy offers to the pernicious habit of "cramming," that we deem it our duty to warn the student against the fatal mistake of neglecting the only true means of becoming proficient in the most important and fundamental department of professional knowledge.

Another novel and essential feature of Mr. Gray's book, is the attention he has bestowed upon regional or surgical anatomy. The relations of arteries are very fully given, and ample directions afforded for the operation of securing them by ligature. Tracheotomy and Lithotomy are treated of in connection with the descriptive anatomy of the neck and perineum. The muscles are considered in their surgical bearing, and the various displacements caused by their action in fracture, illustrated and described. The operations practiced for the division of tendons and muscles, likewise receive their full share of attention. In fact, the whole of this portion of the book is replete with interesting, practical information. The practice of interspersing a work on descriptive anatomy with surgical matter, is sometimes condemned as useless and improper. With such an opinion we cannot agree. The two branches are, it is true, essentially distinct, each demanding separate attention and investigation; but the connection between many parts of anatomy and surgery is so intimate and apparent, that they can nowhere be so successfully studied as during the progress of a dissection. The habit of exhibiting the facts of anatomy in their practical applications is of essential service; also, in refreshing the mind of the student wearied with dry details, and by convincing him of the importance of his subject, it tends to stimulate him to increasing effort.

In conclusion, we heartily commend the work of Mr. Gray to the attention of the medical profession, feeling certain that it should be regarded as one of the most valuable contributions ever made to educational literature.

